# Porcine circovirus type 1 was undetected in vaccine but could be cultured in the cell substrate of Lanzhou lamb rotavirus vaccine

**DOI:** 10.1099/jgv.0.000875

**Published:** 2017-11-22

**Authors:** Qingchuan Yu, Yan Liu, Jialiang Du, Yueyue Liu, Lili Zhang, Tai Guo

**Affiliations:** Division of Enteric Viral Vaccines, National Institutes for Food and Drug Control, Beijing 100050, PR China

**Keywords:** Porcine circovirus, Adventitious agents, Rotavirus vaccine, Infectivity study, Safety, LLR

## Abstract

In 2010, Rotarix was found to be contaminated with infectious porcine circovirus type 1 (PCV1). In China, the Lanzhou lamb rotavirus (LLR) vaccine is the only vaccine used to prevent rotavirus disease. From 2006 to September 2014, more than 54 million doses of LLR vaccines have been lot released. It is a safety issue whether PCV1 is present in the LLR vaccine. Although the cell substrate of LLR, bovine kidney (BK), is different from that of Rotarix, we have investigated the cell’s permissivity for PCV1 by both infectivity and full-length PCR analysis. We have assessed the LLR using a quantitative PCR (qPCR) assay. A total of 171 random batches of LLR final products over a period of 5 years were tested, and no PCV1 was detected (0/171). Infectivity studies showed that two strains of PCV1, the PCV1-prototype, which was derived from PK-15 cells, and the mutant, PCV1-GSK, which was isolated from Rotarix, were capable of replicating in BK cells over a wide m.o.i. ranging from 10 to 0.01. After culture for 6 days, copies of PCV1-prototype DNA were higher than those of PCV1-GSK on average. The genome of the virus was detected at 6 days post-infection. In summary, the LLR vaccine is free of PCV1. Nevertheless, because PCV1 can replicate in the BK cell substrate, manufacturers need to be vigilant in monitoring for this adventitious agent.

## Introduction

In 2010, porcine circovirus type 1 (PCV1) was discovered in Rotarix [1]. While only nucleic acid of PCV1 and PCV2 was found in RotaTeq without intact PCV virions, infectious PCV1 was shown to be present in Rotarix [2]. The US Food and Drug Administration (FDA), vaccine manufacturers, and academic scientists confirmed the original results and investigated the source of contamination. It turned out that the Vero cell lines used to manufacture the vaccine were contaminated with porcine circovirus (PCV) [3] and arose most likely from the porcine trypsin used in the propagation of the Vero cells. PCV was first described as an unexpected contamination in the PK-15 (porcine kidney cells) cell line [4]. There are two types of PCV: PCV1 and porcine circovirus type 2 (PCV2). PCV2 causes post-weaning multisystemic wasting syndrome (PWMS) in swine, while PCV1 has not been shown to cause any porcine disease [5]. PCV DNA has been detected in 5 % of stool samples from US adults [1]. In another study which investigated whether PCV2 sequences could be detected in the stool of recipients of RotaTeq (which is contaminated with PCV2 DNA), 235 out of 826 samples (28.5 %) from 59 vaccine recipients were positive for PCV2 DNA [6]. However, there was no evidence that this DNA shedding was associated with viable PCV. Neither PCV1 nor PCV2 is known to be pathogenic in humans [1, 7, 8].

Nevertheless, PC?V is a novel adventitious agent that needs to be considered by the vaccine industry, especially since PCV infection is distributed worldwide in swine [9–12]. Unintentional introduction of adventitious agents is a recognized potential concern due to their exposure to animal-derived raw materials, such as porcine trypsin. In addition, PCV is highly resistant to many widely used inactivation procedures [13, 14] and thus the risk of contamination is raised. These two rotavirus vaccines – Rotarix and RotaTeq – are widely used worldwide but not in China. In China, the LLR vaccine is produced in BK cells. From 2006 to September 2014, more than 54 million doses of the LLR vaccine have been lot released [15]. Considering the similarity of the attenuated live vaccines and the manufacture processes among the rotavirus vaccines, an investigation for PCV1 in the BK cell substrate was warranted. However, there are differences between China’s LLR vaccine and the other vaccines. The Vero cell lines used to produce Rotarix and RotaTeq are consistent batch-to-batch and easier to monitor because of the stability of the cell substrates. In contrast, BK cells are prepared with fresh bovine kidney (BK) cells and new cells are required for each batch of vaccine. Thus, the potential for contamination is increased and it is important to investigate whether the PCV1 was cultured in the BK cell. Consequently, the LLR final products should be monitored for PCV1 at the same time. In this study, we focused on detection of PCV1.

## Results

### DNA sequencing and**blast** results

The full-length genomes of two PCV1 strains were amplified by PCR. The full-length amplified genomes of PCV1 were sequenced to confirm the identity. blast results showed that the PCV1-prototype was the same genome as that previously reported for PCV1 in PK-15 cells (GenBank accession number: JN133303.1), while the genome of PCV1-GSK was identical to the PCV1 in Rotarix (GenBank accession number: HM143844.1). There are eight nucleotide differences between the two sequences.

### Results of infectivity studies

**Fig. 1.**
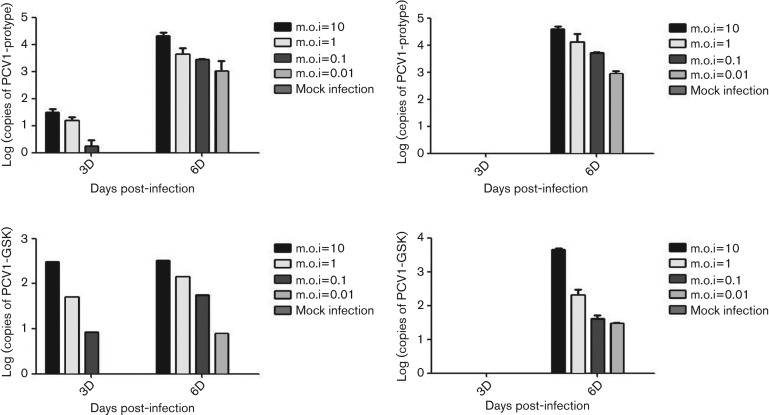
PCV1 quantification results after the infectivity assay on BK cells. The PCV1-prototype group (a and b) and the PCV1-GSK group (c and d) were pretreatedwith d-glucosamine (a and c) or not (b and d) before inoculation. m.o.i. ranges from 10 to 0.01 with a mock-infection group which remained negative throughout.

Results of infectivity assay are shown in Fig. 1. The mock-infection groups (without virus inoculation) were PCV1 negative. After 6 days of culture, the experimental groups were PCV1 positive with or without d-glucosamine pretreatment. However, pretreatment of d-glucosamine (Fig. 1a, c) significantly enhanced the viral replication at 3 days post-infection (p.i.) compared with the group without pretreatment (Fig. 1b, d). According to the data 6 days p.i., positive signals were detected from the infection with 106 copies ml^−1^ (m.o.i.=10) and 103 copies ml^−1^ (m.o.i.=0.01), which means that a small quantity of PCV1 could be enough to infect the BK cells. It is worth noting that using the same copies of PCV1 to infect the BK cells, the replication ratio differed between the two strains of PCV1. At 6 days p.i., copies of the PCV1-prototype are 13.3-fold (m.o.i.=10), 40.6-fold (m.o.i.=1), 85.3-fold (m.o.i.=0.1) and 50.7-fold (m.o.i.=0.01) higher than those of PCV1-GSK.

**Fig. 2.**
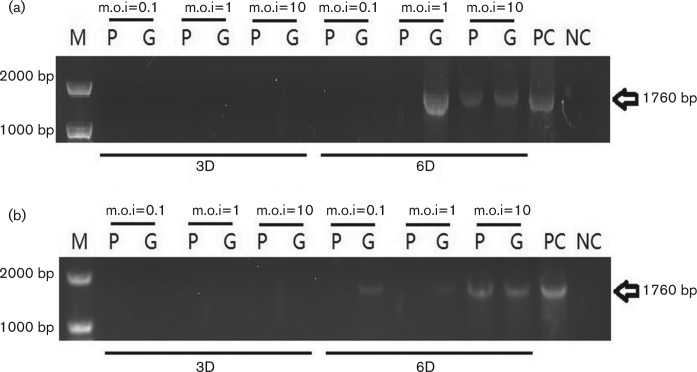
PCV1 full-length amplification results after the infectivity assay on BK cells. (a) Without d-glucosamine pretreatment. (b) With d-glucosamine pretreatment. P and G represent the PCV1-prototype virus and the PCV1-GSK virus, respectively. m.o.i. and sampling time points are marked above and below. PC and NC represent the positive and negative controls.

The genome of PCV1 was detected 112 with full-length primers. The 3 days p.i. samples were negative results while the 6 days p.i. were partly positive (Fig. 2).

### Results of LLR vaccine surveillance

From 2010–2012 and 2014–2015, 171 batches of the LLR vaccine were chosen at random. All these vaccines were stored at 2~8 °C as required. Intact vaccine packages were confirmed before use. The whole DNA was extracted and tested by qPCR methods as described above. All of these vaccine batches showed negative results for PCV1. Batch number information is listed in Table S1 (available with the online version of this article).

## Discussion

Over the years, the presence of adventitious agents in vaccines has been a recurring problem. From SV40 in polio vaccines [(16] and bacteriophages in live viral vaccines [17, 18], to reverse transcriptase activity in measles and other vaccines [19], and PCV in rotavirus vaccines [1]. It is not clear whether these adventitious agents are potential or real threats to human health. According to subsequent studies, PCV1 infects not only porcine source cells, but also cells from other species, such as the Vero cells in the recent incident, which indicates that PCV1 is capable of using more than one kind of cell as a host. GlaxoSmithKline (GSK) investigated Rotarix extensively and the materials used at different stages of the production process. These assessments found the presence of PCV1 DNA in master and working cellular banks, as well as master and working viral seed stocks. Infectivity studies showed that PCV1 sequences in Rotarix bulks and final products were infectious. Although final products of GSK’s other live viral vaccines were shown to be clear of PCV1, the inactivated poliovirus vaccine (IPV) harvest tested positive for PCV1, an unexpected contamination derived from the same cell bank as that used to produce Rotarix [20]. Nevertheless, no infectious virus was detected in IPV bulks. It was concluded that the source of PCV-1 contamination was non-irradiated trypsin that was used in the mid-1990s to manufacture the Vero cell banks [20]. Once passaged cells are contaminated by the infectious and intact PCV1, which persists without causing any visible cell changes, it is almost impossible to eradicate these agents.

PCV was reported as an unexpected contamination in the PK-15 (porcine kidney cells) cell line [4], and thus we screened PCV1 in materials for cell culture in our laboratory to avoid confusing results before we started infectivity studies. Samples from the culture medium, fetal bovine serum (FBS), trypsin (for cell culture and rotavirus activation, porcine-derived and bovine-derived), l-glutamine, penicillin and streptomycin, PBS and HEPES were tested and were found to be PCV1-free (data not shown). RD (human rhabdomyosarcoma cells), MA104 (African green monkey kidney cells), HEK293 (human embryonic kidney cells), CHO-K1 (Chinese hamster ovary cells), Vero (African green monkey kidney cells) and MRC-5 (human fetal lung fibroblast cells), which are widely used for vaccine production and in quality control assays, were also used in infectivity studies along with BK cells. PCV1 replicated significantly in Vero and BK cells while the other cell lines involved could not support PCV1 replication (data not shown), suggesting that PCV1 has a restricted host range. However, our results indicated that both strains of PCV1 have the ability to spread across species: from PK-15 to Vero (pig to monkey) and to BK (pig to cattle). The capacity of PCV1 to cross host species brings new concerns to the vaccine industry. Finding that PCV1 is able to infect CD4+, CD8+, CD14+, CD19+ and CD56+ human cells [21], and the observation of productive PCV1 infection in a subclone of a human hepatocellular carcinoma cell line [22] suggests potential risks for humans. The consequence of long-term contact with live PCV1 remains uncertain.

In our study, two strains of PCV1 were found to be able to infect BK cells within a range of m.o.i. from 10 to 0.01. This indicates the permissivity of BK cells for PCV1 and also demonstrates that PCV1 could be a contaminant in specific cells. Although the virus needs some special conditions (e.g. d-glucosamine) for optimal infection and replication, previous studies demonstrate that some commonly used substances (e.g. NH4Cl) might be able to achieve the same function [23]. Even without d-glucosamine being used for PCV12 infection *in vitro*, after 6 days of cultivation the genome of PCV was detected in infected BK cells, suggesting the possible formation of the progeny virus. d-glucosamine accelerated the replication of virus at 3 days p.i. but did not increase the total amount of virus at 6 days p.i. However, the toxicity of d-glucosamine on low passage cells might affect the ratio of cell survival and decrease the virus production.

The BK cells used to produce the LLR vaccine are different from Vero cells. BK cells are only used within a finite period and then changed for the next batch of the LLR vaccine, which has advantages and disadvantages. Changing the production cells allows less time for PCV1 to adapt to the heterogeneous cells and decreases the possibility of permanent contamination. Using immortalized cells such as Vero, the vaccine manufacturing process might have an increased chance of being exposed to such an adventitious agent. Once established, though, the master cell bank is not changed during manufacture. This brings better consistency not only for vaccine quality, but also for the control of adventitious agents. In contrast, control of BK cells consumes much more energy because the cells are frequently changed. Thus, prevention and detection of adventitious agents are challenging.

The introduction of new technologies makes it possible to detect agents that we were unable to detect before, such as the product-enhanced reverse transcriptase (PERT) assay [19] for agents with transcriptase activity, and microarrays and high-throughput sequencing [1] for other potential contaminants. The virus itself does not remain stable genomically. The PCV1-GSK strain, compared with the PCV1-prototype, has eight point mutations, which might cause attenuation or adaption to the BK cells. Certainly, the PCV1-replicating capacity will require further study. The contaminated Vero cells and the vaccine final product might need to be cleansed of PCV1. Q Sepharose Fast Flow chromatography has been investigated with promising results [24]. Both the discovery of new adventitious agents and the solution of problems bring demands for development of technology.

We have monitored the LLR vaccine over 5 years, including 171 batches. All of these rotavirus vaccine batches were PCV1 negative, suggesting the safety of the LLR vaccine with respect to PCV1 contamination. This may partly be a consequence of the cells used and of conventional monitoring. However, the production process of the vaccine is complicated, containing animal sera and other raw biological materials. The use of BK cells as the cell substrate for vaccine production requires attention to PCV1 control. Using PCV1 test is recommended in multiple steps of LLR vaccine production and in the screening of raw materials. Long-term surveillance is still essential.

## Conclusion

Although the BK cells are susceptible to PCV1, we have shown that 171 batches of the LLR vaccine are free from PCV1 contamination over 5 years. We should strengthen our monitoring and incorporate the PCV1 test item as routine in the future to ensure the safety of vaccines and the health of recipients.

## Methods

### Cells

BK cells were courtesy of Lanzhou Institute of Biological Products (LIBP, Lanzhou City, Gansu Province, PR China). Vero cells (African green monkey kidney; CCL-81) were obtained from the American Type Culture Collection (ATCC, Manassas, VA, USA).

BK cells were grown in minimum essential medium (MEM, Invitrogen) supplemented with 10 % FBS and 100 U ml^−1^ of penicillin and 100 µg ml^−1^ of streptomycin. The second and third passages were used in vaccine manufacture of LLR, so the BK cells were confined into the first four passages in our study. The Vero cell line was cultured in Dulbecco’s modified Eagle’s medium (DMEM, Invitrogen) supplemented with 10 % fetal bovine serum (FBS, Invitrogen), 10 µM HEPES (ThermoFisher) and 100 U ml^−1^ of penicillin and 100 µg ml^−1^ of streptomycin (Thermo Fisher). Both of these cells were grown at 37 °C in a 5 % CO2 atmosphere. BK cells were passaged at a ratio of 1 : 3 every 3 days using trypsin-EDTA (0.05 % trypsin, 0.02 % EDTA-4Na; Invitrogen) while the Vero cell lines were passaged at a ratio of 1 : 8 every 6 days with the same reagents.

### Virus

Two PCV1 strains were used in our study. The PCV1-prototype was isolated from the PK-15 cell line, which was cultured as recommended by the suppliers. The prototype virus was harvested by freeze-thawing three times when the PK-15 cells reached 100 % confluence in flasks. PCV1-GSK was isolated from the Rotarix vaccine as described below. The vaccine was concentrated using a 100 000 MW centrifugal filter (Merck Millipore) and washed with DMEM (FBS-free) to increase the titre and decrease the osmotic pressure caused by the sucrose in the vaccine. The concentrated suspension was heated at 70 °C for 1 h to inactivate rotavirus. One milliliter of virus suspension with an amount of about 10 times of the end product vaccine was applied to Vero cells in 25 cm^2^ flasks at 48 h after cell passaging and inoculated for 2 h in 37 °C atmosphere. The supernatant was discarded, the cells were washed three times with PBS, and 10 ml FBS-free DMEM was added to the flask as a maintenance medium. Vero cells were freeze-thawed three times at 4 days p.i. The viral lysates were centrifuged at 5000 ***g*** for 30 min to harvest the supernatant for the next inoculation process using the same procedure as described above. The titres of both the PCV1-prototype and PCV1-GSK were determined using a qPCR assay as described below.

### DNA preparations

Total cell DNA was prepared from cell pellets using QIAamp DNA Blood Mini Kit (Qiagen) following the manufacturer’s instructions. DNA preparation of LLR vaccine-infected cells was similar. A 200 µl sample was applied to the preparation, and 50 µl DNA was extracted, enabling the original titre of PCV1 to be calculated after the quantification. Prepared DNA was tested with NanoPhotometer (IMPLEN) and samples of A260/A280 ratio ranging from 1.80 to 2.00 were used in the PCR and qPCR assays.

### PCR and qPCR assays

The primers used to amplify the full-length PCV1 genome for sequencing are listed in Table 1. The full-length PCR reaction conditions were as follows: 95 °C for 5 min, followed by 40 cycles of 95 °C for 30 s, 48 °C for 1 min, 72 °C for 2 min, and terminated by 72 °C for 10 min. The qPCR assay was performed using primers and a probe directed at the replicase gene of PCV1, which are shown in Table 1. Reaction conditions for qPCR were as follows: 50 °C for 2 min, 95 °C for 10 min, followed by 40 cycles of 95 °C for 15 s, 60 °C for 1 min. The PCR and qPCR methods have been described previously [2]. PCR assays were done in a Veriti 96 Well Thermal Cycler (Applied Biosystems) while qPCR assays were done in a 7500 Real Time PCR System (Applied Biosystems).

**Table 1.** Primers and the probe used to amplify the full-length and quantitative PCV1

Name	Direction	Sequence	Position
PCV1-full length	Forward	GCATGGATCCATCACTTCGTAATGGT	1010–1035
	Reverse	GCATGGATCCAAAAAAGACTCAGTAA	1035–1010
PCV1-RTPCR	Forward	TGGCCCGCAGTATTTTGATT	785–804
	Reverse	CAGCTGGGACAGCAGTTGAG	856–838
	Probe	FAM-CCAGCAATCAGGCCCCCCAGGAAT-TAMRA	806–829

### PCV1 sequencing and analysis

The amplified full-length fragments of PCV1 were analysed by agarose gel electrophoresis. The gel bands of approximately 1760 bp were excised and purified with QIAquick Gel Extraction Kit (Qiagen). The products were cloned into pEASY-T3 Vector (Transgene) and transformed into competent cells (Transgene) according to the manufacturer’s instructions. Plasmids extracted from bacteria were analysed by ABI 3730 DNA Analyzer (Applied Biosystems) using M13 sequencing primers. Sequence analysis was done using nucleotide blast.

### PCV1 infectivity studies

BK cells were passaged and counted. Cell suspensions were diluted to 105 cells ml^−1^ and cultured in 12-well plates, 1 ml well^−1^. After culture in 37 °C, 5 % CO2 atmosphere for 48 h, the cells were washed with prewarmed PBS three times. d-glucosamine 300 mM (Sigma-Aldrich) was applied to the cells and incubated in 37 °C for 30 min to promote the replication of PCV1 [25]. FBS-free MEM was used in the control group. The supernatant was extracted and cells were washed with PBS three times. Two strains of PCV1 were serially diluted at a ratio of 1 : 10 to obtain 106 to 103 copies ml^−1^ virus, added to the cells and incubated at 37 °C for 2 h. The mock-infection group was inoculated with FBS-free MEM. Subsequently, cells were washed three times with PBS and FBS-free MEM was added as the maintenance medium. This time point was set as time 0. After 3 and 6 days incubation, the cells were freeze-thawed three times to release the viruses. The viral samples were then purified to extract total DNA which was used for in PCR and qPCR.

### Detection of PCV1 in LLR

Sampling of the LLR vaccine from 2010–2012 and 2014–2015 was performed on samples selected at random. DNA of each batch was purified and tested by the qPCR assay. The results were analysed using 7500 Software 2.0.5 (Applied Biosystems).

## Supplementary Data

191 Supplementary File 1
