# Supplementary material for: Porcine circovirus type 1 was undetected in vaccine but could be cultured in the cell substrate of Lanzhou lamb rotavirus vaccine
Source: J Gen Virol. 2017 Nov 22;99(1):103–8. doi: 10.1099/jgv.0.000875 (PMC5882086; doi:10.1099/jgv.0.000875)
Supplement: Supplementary File 1 [file jgv-99-103-s001.pdf]

Supplementary table 1. Batch number of vaccines detected

Year	2010	2011		2012			2014	2015	
Batch number	201006084	201101001	201109106	201200469	201203046	201204083	201407105	201501023	201511107
	201008107	201101003	201109111	201201002	201203047	201204084	201409116	201501026	201511108
	201008108	201101006	201109112	201201003	201203048	201204085	201411146	201501027	201511109
	201008109	201101007	201109114	201201005	201203049	201205087	201411147	201504035	201511113
	201008110	201101008	201109116	201201006	201203051	201205088	201411148	201504036	201511116
	201008113	201103009	201110117	201201007	201203058	201205091	201412149	201504037	201511117
	201008114	201103010	201110121	201201015	201203059	201205092	201412150	201504038	201511118
	201008115	201103012	201110132	201201016	201203060	201205094	201412156	201504040	201511119
	201008116	201103014	201112073	201201018	201203062	201205096	201412157	201504041	201511120
	201009120	201103019	201112158	201202024	201203066	201205097	201412159	201504052	201511121
	201009121	201104020	201112160	201202025	201204042	201205102	201412163	201506076	201511122
	201009127	201108092	201112164	201202026	201204069	201205103	201412164	201506076	201511123
	201010129	201108095	201112165	201202029	201204070	201205106		201506077	201511124
	201010131	201108096	201112166	201202035	201204073	201207140		201506078	201511125
	201010133	201108098	201112167	201202037	201204075	201209169		201511102	201511126
	201012134	201109102	201112170	201202038	201204076	201209171		201511103	201512127
	201012137	201109103	201112171	201202039	201204077	201209173		201511104	201512128
	201012138	201109105	201112176	201202040	201204078	201209174		201511105	201512129
	201012139			201202041	201204079	201209176		201511106	201512130
	201012145			201202042	201204080	201210187			
				201202043	201204081	201212140			
				201202044	201204082				
